# Association of a novel circulating tumor DNA next-generating sequencing platform with circulating tumor cells (CTCs) and CTC clusters in metastatic breast cancer

**DOI:** 10.1186/s13058-019-1229-6

**Published:** 2019-12-04

**Authors:** Andrew A. Davis, Qiang Zhang, Lorenzo Gerratana, Ami N. Shah, Youbin Zhan, Wenan Qiang, Brian S. Finkelman, Lisa Flaum, Amir Behdad, William J. Gradishar, Leonidas C. Platanias, Massimo Cristofanilli

**Affiliations:** 1 Robert H. Lurie Comprehensive Cancer Center, Feinberg School of Medicine, Northwestern University, Chicago, IL USA; 2 Department of Medicine, University of Udine, Udine, UD Italy; 3 Department of Pathology, Northwestern University, Chicago, IL USA; 4 Department of Medicine-Hematology and Oncology, Robert H Lurie Comprehensive Cancer Center, Feinberg School of Medicine, 710 N. Fairbanks Court- Olson Pavilion, Suite 8-250A, Chicago, IL 60611 USA

**Keywords:** ctDNA, NGS, CTCs, CTC clusters, MBC

## Abstract

**Purpose:**

Liquid biopsies, including circulating tumor DNA (ctDNA) and circulating tumor cells (CTCs), can be used to understand disease prognosis, tumor heterogeneity, and dynamic response to treatment in metastatic breast cancer (MBC). We explored a novel, 180-gene ctDNA panel and the association of this platform with CTCs and CTC clusters.

**Methods:**

A total of 40 samples from 22 patients with MBC were included in the study. For the primary analysis, all patients had ctDNA sequencing using the PredicinePLUS™ platform. CTCs and CTC clusters were examined using the CellSearch™ System. Clinical and pathological variables were reported using descriptive analyses. Associations between CTC count and specific genomic alterations were tested using the Mann-Whitney *U* test.

**Results:**

Of 43 sequenced patients, 40 (93%) had at least one detectable genomic alteration with a median of 6 (range 1–22). Fifty-seven different genes were altered, and the landscape of genomic alterations was representative of MBC, including the commonly encountered alterations *TP53*, *PTEN*, *PIK3CA*, *ATM*, *BRCA1*, *CCND1*, *ESR1*, and *MYC*. In patients with predominantly hormone-receptor-positive MBC, the number of CTCs was significantly associated with alterations in *ESR1* (*P* < 0.005), *GATA3* (*P* < 0.05), *CDH1* (*P* < 0.0005), and *CCND1* (*P* < 0.05) (Mann-Whitney *U* test). Thirty-six percent of patients had CTC clusters, which were associated with alterations in *CDH1*, *CCND1*, and *BRCA1* (all *P* < 0.05, Mann-Whitney *U* test). In an independent validation cohort, CTC enumeration confirmed significant associations with *ESR1* and *GATA3*, while CTC clusters were significantly associated with *CDH1*.

**Conclusions:**

We report on a novel ctDNA platform that detected genomic alterations in the vast majority of tested patients, further indicating potential clinical utility for capturing disease heterogeneity and for disease monitoring. Detection of CTCs and CTC clusters was associated with particular genomic profiles.

## Introduction

Liquid biopsies have emerged as clinical tools for prognostication, molecular analysis, and detection of genomic alterations in blood [1]. The most well-studied components of liquid biopsies, circulating tumor cells (CTCs) and circulating tumor DNA (ctDNA), give insight into the “liquid phase” of solid tumors by providing information on the spatial and temporal heterogeneity of metastatic breast cancer (MBC). Applications with potential clinical utility are being explored, including detection of minimal residual disease, dynamic treatment monitoring, and disease resistance [2, 3]. In MBC, prior work demonstrated that CTC detection and enumeration defined two subgroups of patients, stage IV_indolent_ (< 5 CTCs per 7.5 mL of blood) and stage IV_aggressive_ (≥ 5 CTCs) [4, 5]. This staging has been previously validated to stratify patients into two predefined cohorts with dramatically different prognostic outcomes, regardless of tumor type, site of disease, or line of therapy. Understanding the genomic changes that define these cohorts of patients is critical.

In comparison to CTCs, CTC clusters, which consist of aggregates of two or more cells, are encountered more rarely in the circulation. However, these cell groupings are associated with high metastatic potential, particular DNA methylation patterns, and potentially poor prognosis [6–8]. Initial studies have explored the cellular and genetic makeup of these cell groupings, but much remains unknown regarding how these clusters form and the genetic changes that contribute to the mechanisms of how these groupings metastasize in breast cancer [9].

In MBC, ctDNA genomic alterations have been studied to understand the genetic heterogeneity of tumor resistance. Specifically, markers of endocrine resistance emerge in response to the selective pressures of endocrine therapy with either tamoxifen or aromatase inhibitors as single agents or in combination with CDK4/6 inhibitors in MBC [10]. These events lead to clonal selection and emergence of specific genomic alterations, such as in *RB1*, *PIK3CA* driver mutations, and new *ESR1* mutations. Furthermore, early changes in *PIK3CA* ctDNA can predict progression-free survival for patients treated with palbociclib and fulvestrant [11]. The impact of novel sequencing ctDNA panels in terms of longer DNA sequencing length and a greater number of genomic alterations on detection of resistance mutations in MBC is unknown.

Here, we explore the interplay between CTCs, CTC clusters, and ctDNA genomic alterations in a cohort of patients with predominantly hormone-receptor-positive (HR+) MBC. For CTC detection and enumeration, we utilized CellSearch™, and for ctDNA detection, we used the PredicinePLUS™ 180-gene panel with independent validation that was performed using Guardant360. These studies explore the potential utility of these platforms and how to integrate this information with other liquid-biopsy-derived biomarkers. We demonstrate the analytical validity of this novel ctDNA platform and the association of CTCs and CTC clusters with particular mutational profiles.

## Methods

### Patient selection and study design

The Institutional Review Board (IRB) at the Robert H. Lurie Comprehensive Cancer Center at the Northwestern University Feinberg School of Medicine approved the study. Informed consent from patients was waived per the IRB. The study was performed in concordance with the Health Insurance Portability and Accountability Act. Forty-nine samples were initially evaluated. Six samples had cfDNA yield less than 5 ng and, therefore, did not pass quality control, based on having less than 90% of regions with greater than 3000X coverage. In total, 40 of 43 (93%) passed next-generation sequencing (NGS) quality control for sequencing. As a result, the final cohort consisted of 40 samples from 22 patients with MBC with all samples collected between 2016 and 2017. Sequencing was performed in two batches in March and June 2018.

A cohort of patients with MBC with CTCs and CTC clusters were identified for ctDNA sequencing. CTCs were obtained under a prospective Investigator Initiated Trial (IIT) (NU16B06) at the Robert H. Lurie Comprehensive Cancer Center at Northwestern University (Chicago, IL, USA). CTC collection was performed at baseline prior to initiation of the next line of treatment.

### CTC detection and enumeration

CTC analysis was performed using the CellSearch™ System (Menarini Silicon Biosystems, PA, USA). Approximately 10 mL of whole blood was collected into CellSave Stabilizing Tubes and processed via Celltracks Autoprep. Immunomagnetic sorting was used to characterize epithelial cell adhesion molecule (EpCAM)-positive, pan-cytokeratin (CK)-positive, DAPI-positive, and CD45-negative cells. Cells were reviewed via the Celltracks Analyzer II. CTC clusters were characterized as an aggregation of at least two cells with a distinct nuclei and an intact cytoplasm membrane.

### ctDNA sequencing

Patients with 10 mL of whole blood specimens had ctDNA sequencing from plasma performed using the PredicinePLUS™ platform (Predicine, Inc., Hayward, CA, USA). The platform includes a 180-gene panel with 565 kb of sequencing coverage including single nucleotide variants (SNVs), copy number variants (CNVs), and a total of 88 fusion genes (Additional file 1: Table S1). cfDNA yield was measured using Bioanalyzer 2100, and sequencing was performed using an Illumina platform with an in-house proprietary bioinformatics pipeline to align ctDNA sequences and to determine genomic alterations. Assay sensitivity was set at a minimum of 0.25% mutant allele frequency (MAF) for all genomic regions and 0.1% MAF for hotspot variants. Targeted sequencing coverage was greater than 20,000X.

Based on a minimum cfDNA input of 15 nanograms (ng), sensitivity for SNVs, CNVs, and gene fusions were reported as 94.4%, 95.0%, and 83.3%, respectively, with positive predictive values (PPV) of 99.7%, 100%, and 100%, respectively. Of note, NGS of these samples was performed for research purposes only. Patients received standard, guideline-based, systemic treatments. For independent validation, NGS was performed on plasma samples in a subset of patients (*N* = 14) with concurrent commercial Guardant360 testing (Guardant Health, Redwood City, CA) [12]. In addition, 84 patients with Guardant360 testing and CTC evaluation were analyzed to confirm associations between CTCs and particular genomic alterations. CTC and ctDNA analyses were linked to a deidentified clinical database.

### Statistical analysis

Clinical and pathological variables were reported using descriptive analyses. Associations between CTC count and specific genomic alterations were tested using the Mann-Whitney *U* test. All analyses were performed using STATA (StataCorp (2015) Stata Statistical Software: Release 14.2 College Station, TX: StataCorp, LP).

## Results

### Patient characteristics

Patient characteristics for the 22 MBC patients included in the cohort are included in Table 1. All patients were female. The sample consisted of 17 HR+, HER2-negative patients, 1 HR-negative, HER2-positive, 1 HR+, HER2-positive, and 3 triple-negative breast cancer patients. There were 18 patients with invasive ductal carcinoma (IDC) and 4 patients with invasive lobular carcinoma (ILC). Median prior to lines of treatment in the metastatic setting was 2 [range 0–7]. In total, 81.8% of patients had bone involvement and 68.2% had visceral disease (Additional file 1: Table S2).

**Table 1:** Patient characteristics

Cohort	
Number of patients	22
Number of collections	40
Sex	
Female	22 (100%)
Pathology	
IDC	18 (81.8%)
ILC	4 (18.2%)
Histologic subtype	
HR+, HER2−	17 (77.3%)
HR−, HER2+	1 (4.5%)
HR+, HER2+	1 (4.5%)
TNBC	3 (13.6%)
Clinical subtype	
IBC	6 (27.3%)
Non-IBC	16 (72.7%)
Prior therapies in metastatic setting	2* [0–7]
Sites of disease	
Bone	18 (81.8%)
Visceral	15 (68.2%)
CTC clusters	
Yes	8 (36.4%)
No	14 (63.6%)
Total blood draws with clusters	14 (35.0%)

*IDC* invasive ductal carcinoma, *ILC* invasive lobular carcinoma, *HR* hormone receptor, *TNCB* triple-negative breast cancer, *IBC* inflammatory breast cancer

*The median

### Platform characteristics

Forty samples from 22 patients were included in the final analyses [range 1–4 samples per patient]. Of 43 initial samples, 40 passed NGS quality control (40/43, 93%) and had detectable genomic alterations (Table 2). Mean, median, and range for number of genomic alterations were 6.7, 6.0, and 1–22, respectively. cfDNA yield ranged from 7.8 to 272.5 ng. MAF of detectable mutations ranged from 0.11 to 68.6%.

**Table 2:** Characteristics of the PredicinePLUS™ platform and detected alterations

Cohort	
Total cases	49
Cases included in final analyses	40
Regions analyzed	180 genes
Panel size	565 kb
Samples with detectable alterations	40/43 (93%)
Number of genomic alterations	
Mean	6.7
Median	6.0
Range	1–22
Number of genes with detected alterations	57
Variant allele frequency of detected alterations	0.11–68.6%
Commonly detected SNV/indels	*TP53*, *PTEN*, *PIK3CA*, *ATM*, *ESR1*
Commonly detected copy number amplifications	*MYC*, *CCND1*, *PIK3CA*
Commonly detected copy number losses	*BRCA1*, *CDKN2A*, *ATM*

*SNV* single nucleotide variant, *Indels* insertion-deletion mutations

### ctDNA genomic alterations

**Fig. 1:**
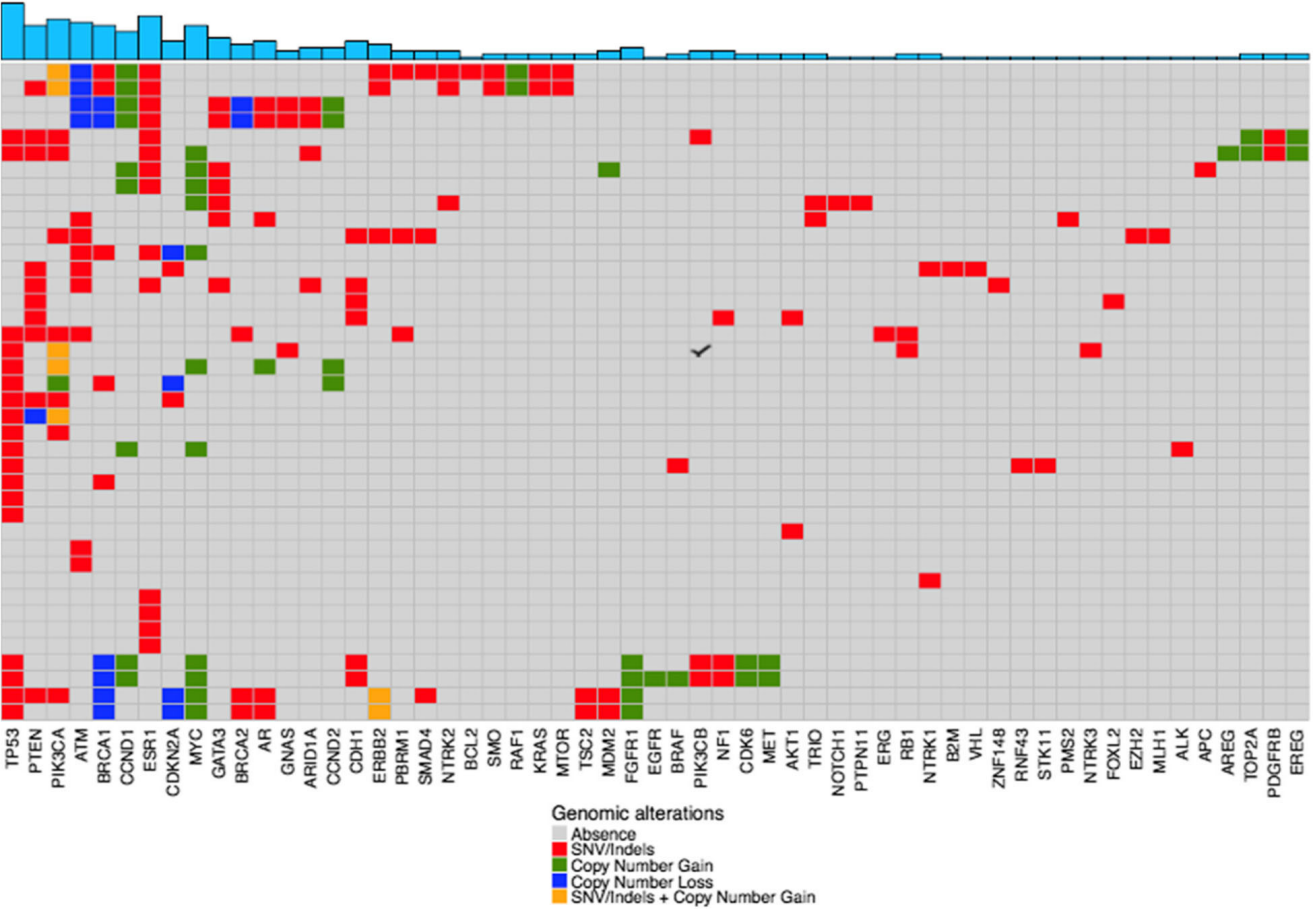
Landscape of genomic alterations. Shown is a heatmap of all detected genomic alterations. Top blue panel indicates the total number of alterations detected for each gene. The colors below indicate the specific types of genomic alterations including SNV/indels (red), copy number gain (green), copy number loss (blue), and SNV/indel + copy number gain (yellow). Each row indicates a sample (*N* = 40) and each column represents a gene

The landscape of genomic alterations in the cohort is included in Fig. 1. Identified alterations were representative of commonly encountered tissue and blood-based NGS alterations including the following: *TP53*, *PTEN*, *PIK3CA*, *ATM*, *BRCA1*, *CCND1*, *ESR1*, and *MYC*. In total, 267 genomic alterations from 57 different genes were reported. Copy number gains were encountered in 16 genes and copy number losses in 5 genes. MAF of common variants are shown in Additional file 1: Figure S1. For patients with concurrent PredicinePLUS™ and Guardant360 testing (*N* = 14), high concordance was observed in orthogonal samples across representative genomic alterations (*ESR1* 92.9%, *PIK3CA* 100%, *MYC* copy number variations 71.4%) (Gerratana L, Zhang Z, Shah A, Davis A, Zhan Y, Qiang W, Finkelman B, Flaum L, Behdad A, Gradishar WJ *et al*: Analytical and clinical performance of a novel next generation sequencing-based (NGS) circulating tumor DNA (ctDNA) platform for the evaluation of samples from metastatic breast cancer (MBC). In. Under Review; 2019).

### Association of CTC enumeration with ctDNA alterations

**Fig. 2:**
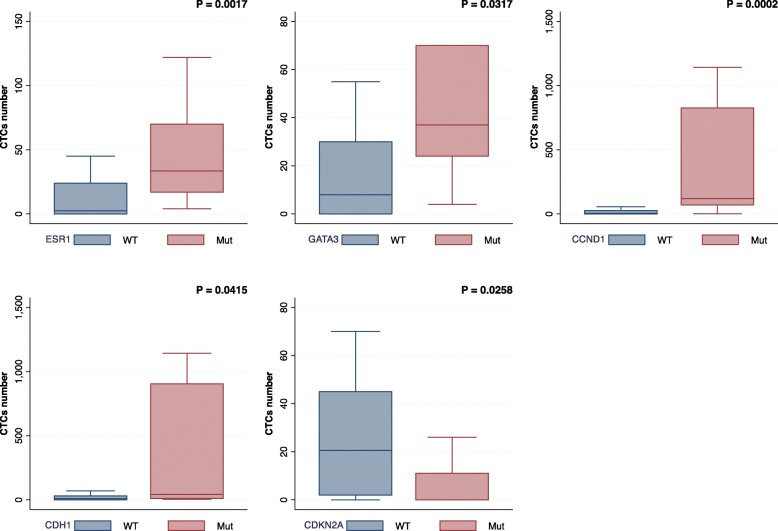
Genomic alterations associated with CTCs. Genomic alterations in *ESR1*, *GATA3*, *CCND1*, and *CDH1* were significantly associated with higher number of CTCs (*P* < 0.05, Mann-Whitney *U* test). In contrast, alterations in *CDKN2A* were more commonly observed in samples with low CTC count (*P* < 0.05, Mann-Whitney *U* test). In the validation cohort, significant associations were confirmed for *ESR1* (*P* < 0.005) and *GATA3* (*P* < 0.05)

Patients were categorized into stage IV_indolent_ (< 5 CTCs per 7.5 mL of blood) and stage IV_aggressive_ (≥5 CTCs) disease based on CTC enumeration. In total, there were 8 patients (36%) with stage IV_indolent_ and 14 patients (64%) with stage IV_aggressive_ at baseline CTC draw in the cohort. Samples with *CDKN2A* alterations had a significantly lower number of CTCs (*P* < 0.05, Mann-Whitney *U* test) (Fig. 2). In contrast, samples with a higher number of CTCs were significantly associated with alterations in *ESR1* (*P* < 0.005), *GATA3* (*P* < 0.05), *CDH1* (*P* < 0.0005), and *CCND1* (*P* < 0.05) with stage IV_aggressive_ disease associated with *ESR1* mutations (Mann-Whitney *U* test). In independent validation using Guardant360 testing of 84 patients, number of CTCs was confirmed to have significant associations with *ESR1* (*P* < 0.005) and *GATA3* (*P* < 0.05), as well as copy number changes in *MYC* (*P* < 0.05). Characteristics of this cohort are included in Additional file 1: Table S3.

### Association of CTC clusters with ctDNA alterations

**Fig. 3:**
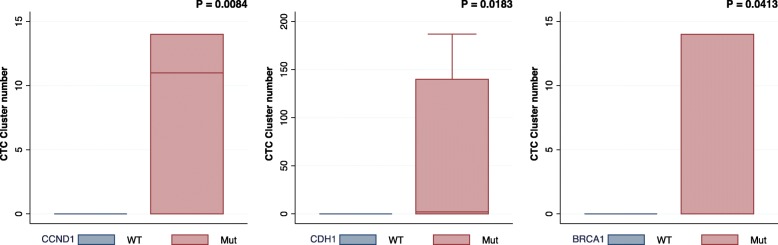
Genomic alterations in patients with CTC clusters. Genomic alterations in *CCND1*, *CDH1*, and *BRCA1* were significantly associated with the number of CTC clusters (*P* < 0.05, Mann-Whitney *U* test). In the validation cohort, a significant association was confirmed for *CDH1* (*P* < 0.005)

**Fig. 4:**
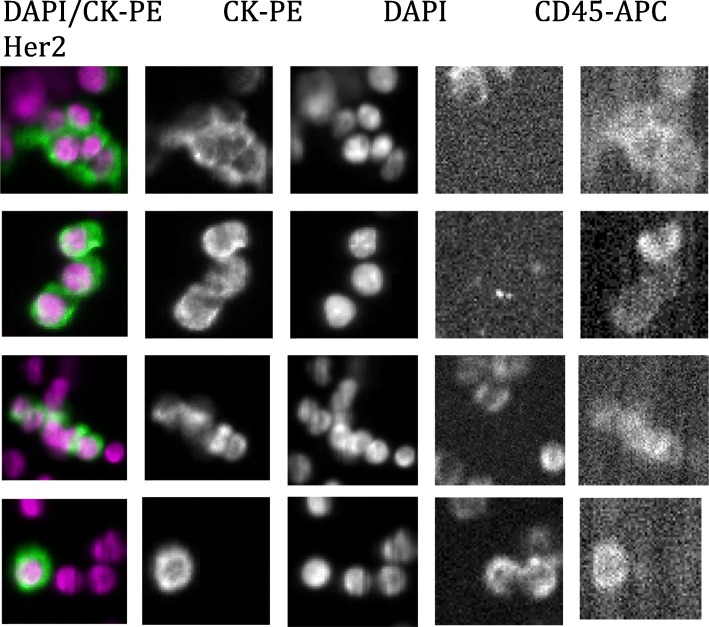
Representative images of a patient with CTC clusters. Shown are representative images of a patient with CTC clusters with nuclear (DAPI) and cytokeratin (CK-PE) staining. CD45 stains for non-CTC leukocytes. HER2/neu staining further distinguished CTCs from leukocytes in this patient. This sample was associated with the following ctDNA genomic alterations: *CDH1*, *TP53*, *NF1*, *PIK3CB*, *BRCA1*, *CCND1*, *CDK6*, *FGFR1*, *MET*, and *MYC*

Eight patients (36%) were found to have CTC clusters in at least one blood collection in the cohort. Furthermore, 12/40 (30%) of total blood draws contained CTC clusters. All patients with CTC clusters had stage IV_aggressive_ disease. CTC clusters were significantly associated with somatic genomic alterations in *CDH1*, *CCND1*, and *BRCA1* (all *P* < 0.05, Mann-Whitney *U* test) (Figs. 3 and 4). In the validation cohort, CTC clusters were significantly associated with *CDH1* (*P* < 0.005).

### Case vignette

We present a case illustrating the potential clinical utility for serial blood monitoring (Additional file 1: Figure S2). The patient was a 67-year-old female with a history of localized HR+ HER2-negative breast cancer treated with lumpectomy and radiation who declined endocrine therapy. About 2 years later, she developed right upper quadrant pain and was found to have metastatic disease to the liver, which was biopsy confirmed HR+ HER2-negative breast adenocarcinoma. She was initiated on single-agent anastrozole for approximately 2 years and subsequently developed bone metastasis in her scapula and lumbar spine. She was changed to fulvestrant (declined palbociclib) and zoledronic acid with progression of disease 4 months later in her liver and bones. At this time, CTC evaluation demonstrated 45 CTCs of which 5 were HER2 positive with 2 CTC clusters present (timepoint 1). ctDNA NGS revealed an *ERBB2* (*HER2*) S310F mutation. Treatment was initiated with trastuzumab and capecitabine. Serial CTC collection 3 months later revealed 0 CTCs (timepoint 2). Eighteen months later, after 22 cycles of trastuzumab, ctDNA revealed increasing clonal heterogeneity with 9 alterations present in blood including 5 different *HER2* mutations and 9 CTCs (1 HER2 positive) (timepoint 3). Subsequent imaging confirmed progression of disease in the liver and lymph nodes.

## Discussion

The clinical potential for liquid biopsies, including CTCs and ctDNA, for prognostication and disease monitoring is expanding. We analyzed a novel, sensitive, 180-gene, 565-kb sequencing platform to analyze and report on the landscape of alterations and association of genomic changes with a cohort of predominantly HR+ MBC characterized by CTC enumeration and CTC clusters. The study demonstrated that using the sequencing length of this platform, 100% of samples that passed quality control and 40 of 43 that underwent sequencing (93%) had at least one somatic alteration detected. This suggests a clear potential for using this technique to detect and monitor dynamic changes in the blood of patients with MBC. In total, 57 genes with genomic alterations were detected including SNVs, indels, and CNVs. Longer sequencing panels may also aid in identifying novel resistance mutations in blood and better represent tumor heterogeneity to eventually capture tumor mutational burden, non-invasively.

We observed that higher number of CTCs was associated with genomic alterations in *ESR1*, *GATA3, CDH1*, and *CCND1*, while lower number of CTCs was associated with *CDKN2A* alterations. In independent validation, these findings were confirmed for *ESR1* and *GATA3*. This supports the relation between CTCs as a marker of poor prognosis and the ability to detect specific resistance mutations (e.g., *ESR1*) with implications for clinical practice. Prior work in our group has demonstrated that *ESR1* mutations in single CTCs matched mutations observed in ctDNA, which demonstrates a mechanism to link CTCs with the release of ctDNA into the blood [13]. Furthermore, alterations in *CDH1*, *CCND1*, and *BRCA1* were associated with a higher number of CTC clusters with the validation cohort confirming *CDH1* as statistically significant. The *CDH1* gene is involved in the production of epithelial cadherin (E-cadherin). Therefore, the gene plays a role in cell adhesion, chemical signaling, and cell movement, all of which may contribute to the metastatic process. Somatic variants in *CDH1* were seen in both ILC and IDC patients in our cohort, although classically this mutation is associated with ILC histology [14]. Further studies are needed to explore this mutation as an indicator of metastatic potential.

## Conclusions

In summary, this novel ctDNA sequencing platform identified genomic alterations in the vast majority of tested patients, reflecting the genomic heterogeneity of patients with predominantly HR+ MBC. This suggests a clear clinical potential for disease monitoring using this platform given the frequency of genomic alterations encountered in our sample. Additional analyses enabled us to characterize particular genomic alterations and different biology based on CTC enumeration and the identification of CTC clusters. Limitations of this study include the relatively small sample size and that some patients with more than one ctDNA sample could have biased the analyses of specific ctDNA alterations. However, these data are consistent with previous experiences using different NGS platforms and were validated in an independent cohort in our study [15]. These findings, therefore, further demonstrate the potential of combining CTCs and ctDNA for comprehensive liquid biopsy analysis to accurately represent genomic heterogeneity and to detect resistance mutations in a non-invasive manner with implications for clinical management of patients with MBC.

## Supplementary information

**Additional file 1 Table S1**. PredicinePLUS™ 180-gene panel. **Table S2**. Treatment data and sites of disease. **Table S3**. Characteristics of Guardant360 validation cohort. **Figure S1**. Mutant allele frequency of 100 most common variants in the cohort. **Figure S2**. Case vignette demonstrating the potential clinical utility of serial liquid biopsy assessment.

## Data Availability

The datasets during and/or analyzed during the current study are available from the corresponding author on reasonable request.
