# Supplementary material for: Association of a novel circulating tumor DNA next-generating sequencing platform with circulating tumor cells (CTCs) and CTC clusters in metastatic breast cancer
Source: Breast Cancer Res. 2019 Dec 4;21:137. doi: 10.1186/s13058-019-1229-6 (PMC6894208; doi:10.1186/s13058-019-1229-6)
Supplement: Supplementary file 1 — Additional file 1 Table S1. PredicinePLUS™ 180-gene panel. Table S2. Treatment data and sites of disease. Table S3. Characteristics of Guardant360 validation cohort. Figure S1. Mutant allele frequency of 100 most common variants in the cohort. Figure S2. Case vignette demonstrating the potential clinical utility of serial liquid biopsy assessment. [file 13058_2019_1229_MOESM1_ESM.docx]

**Table S1: PredicinePLUS^TM^ 180-gene panel**


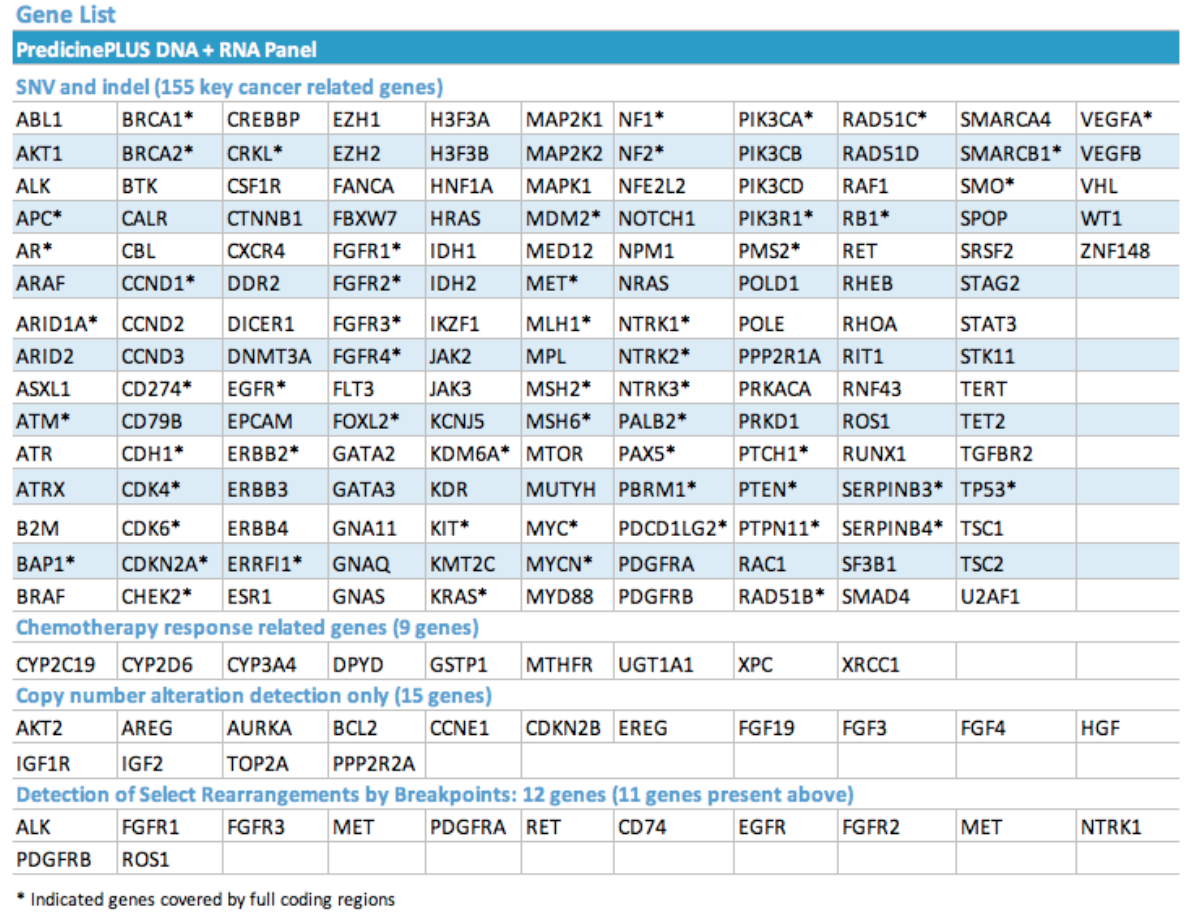


**Table S2: Treatment data and sites of disease**

| Patient | Prior Therapy in Metastatic Setting | Number of Prior Therapies | Sites(s) of Disease |
| --- | --- | --- | --- |
| 1 | capecitabine, docetaxel/carboplatin/trastuzumab/pertuzumab | 2 | liver, lung, bone |
| 2 | palbociclib/letrozole, vinorelbine/capecitabine | 2 | liver, bone, LN, soft/soft tissue |
| 3 | palbociclib/fulvestrant, durvalumab/tremelimumab (clinical trial), ixempra/capecitabine | 3 | liver, bone, LN |
| 4 | cisplatin/radiation (clinical trial) | 1 | bone |
| 5 | none | 0 | skin/soft tissue |
| 6 | fulvestrant, fulvestrant/palbociclib, letrozole, capecitabine | 4 | bone, serosa |
| 7 | carboplatin/paclitaxel, palbociclib/letrozole, liposomal doxorubicin | 3 | lung, LN |
| 8 | carboplatin/abraxane, pembrolizumab/capecitabine (clinical trial) | 2 | bone, LN, lung |
| 9 | anastrozole | 1 | bone, LN |
| 10 | palbociclib/letrozole | 1 | liver, lung, bone |
| 11 | tamoxifen, capecitabine, fulvestrant/everolimus, 5-FU/epirubicin/cyclophosphamide, carboplatin/gemcitabine, abraxane/everolimus/trastuzumab/pertuzumab, eribulin/trastuzumab/pertuzumab | 7 | liver, lung, bone |
| 12 | carboplatin/gemcitabine | 1 | bone, LN, serosa |
| 13 | tamoxifen, palbociclib/letrozole | 2 | lung, bone, serosa |
| 14 | none | 0 | liver, bone, LN |
| 15 | exemestane, docetaxel, everolimus, capecitabine, epirubicin/ixabepilone | 5 | bone, lung, CNS |
| 16 | none | 0 | bone |
| 17 | carboplatin/gemcitabine, paclitaxel, capectabine, doxorubicin, eribulin | 5 | lung, LN, skin/soft tissue |
| 18 | vinorelbine, anastrozole, fulvestrant/palbociclib, everolimus/exemestane | 4 | bone, LN, serosa, CNS |
| 19 | docetaxel/trastuzumab/pertuzumab, T-DM1 | 2 | liver, skin/soft tissue, serosa |
| 20 | adriamycin/cyclophosphamide, carboplatin/paclitaxel, eribulin | 3 | liver, bone, LN, skin/soft tissue |
| 21 | anastrozole, fulvestrant, trastuzumab/capecitabine | 3 | liver, bone |
| 22 | carboplatin/paclitaxel, exemestane, letrozole/palbociclib, capecitabine | 4 | liver, bone, LN, serosa |

LN: lymph node; CNS: central nervous system

**Table S3**: Characteristics of Guardant360 validation cohort

|  | | |
| --- | --- | --- |
| Cohort | |  |
|  | Number of patients | 84 |
| Pathology | |  |
| IDC | | 70 (83.3%) |
| ILC | | 7 (8.3%) |
| Unknown | | 7 (8.3%) |
| Subtype | |  |
|  | Luminal | 36 (42.9%) |
|  | HER2+ | 21 (25.0%) |
|  | TNBC | 27 (32.1%) |
| Clinical Subtype | |  |
|  | IBC | 43 (51.2%) |
|  | Non-IBC | 41 (48.8%) |
| Sites of Disease | |  |
|  | Bone | 39 (46.4%) |
|  | Visceral | 43 (51.2%) |
| CTC Clusters | |  |
|  | Yes | 9 (10.7%) |
|  | No | 75 (89.3%) |

**Figure S1: Mutant allele frequency of 100 most common variants in the cohort**
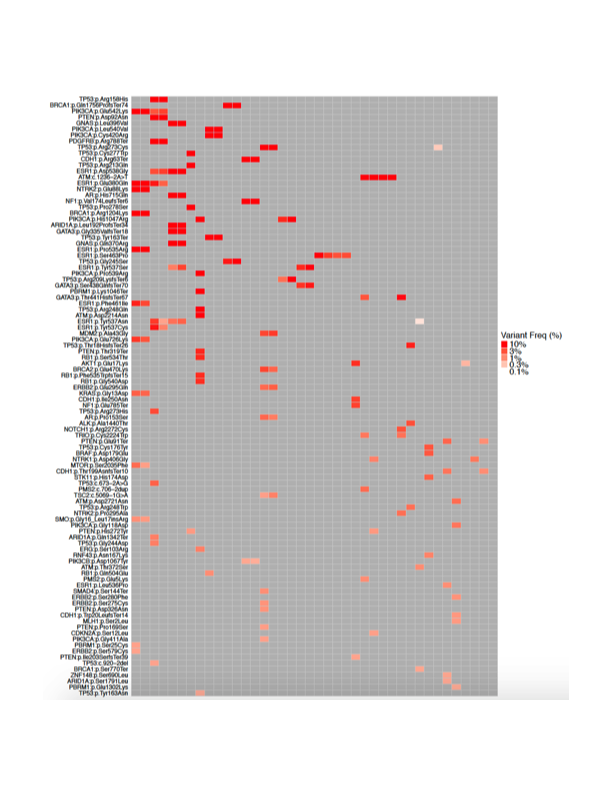


**Figure** S**2: Case vignette demonstrating the potential clinical utility of serial liquid biopsy assessment**

**Association of a novel circulating tumor DNA next-generating sequencing platform with circulating tumor cells (CTCs) and CTC clusters in metastatic breast cancer**

Andrew A. Davis^1^, Qiang Zhang^1^, Lorenzo Gerratana^1,2^, Ami N. Shah^1^, Youbin Zhan^1^, Wenan Qiang^1^, Brian S. Finkelman^1,3^, Lisa Flaum^1^, Amir Behdad^1,3^, William J. Gradishar^1^,

Leonidas C. Platanias^1^, Massimo Cristofanilli^1^

^1^Robert H. Lurie Comprehensive Cancer Center, Feinberg School of Medicine, Northwestern University, Chicago IL;

^2^Department of Medicine, University of Udine, Udine, UD, Italy

^3^Department of Pathology, Northwestern University, Chicago, IL
